# Pandemic and post-pandemic shifts in onset and severity of pediatric nephrotic syndrome: a comparative cohort analysis

**DOI:** 10.3389/fimmu.2026.1860961

**Published:** 2026-08-26

**Authors:** Elena Jechel, Ingrith Miron, Cristina Gavrilovici, Iuliana Magdalena Starcea, Ancuta Lupu, Adriana Mocanu, Otilia Elena Frasinariu, Tudor Ilie Lazaruc, Tania Rusu, Maria Oana Sasaran, Ruxandra Russu, Oana Raluca Temneanu, Alin Horatiu Nedelcu, Ionela Daniela Morariu, Emil Anton, Vasile Valeriu Lupu

**Affiliations:** 1 Grigore T. Popa University of Medicine and Pharmacy, Iasi, Romania; 2 Regina Maria Healthcare Campus, Iasi, Romania; 3 Faculty of Medicine, “George Emil Palade” University of Medicine, Pharmacy, Science and Technology, Targu Mures, Romania

**Keywords:** child, corticosensitivity, COVID-19, infections, pediatric nephrotic syndrome, predictive factors

## Abstract

**Background:**

Pediatric idiopathic nephrotic syndrome (INS) involves complex immunological mechanisms, and clinical presentation may be influenced by environmental factors and access to medical services. The impact of the COVID-19 pandemic on its clinical and evolutionary profile remains insufficiently characterized.

**Objectives:**

To compare pandemic and post-pandemic pediatric INS regarding demographic, clinical and biological characteristics and early disease evolution, and to explore factors associated with infection-related onset and corticosteroid response.

**Methods:**

Single-center retrospective study on 59 pediatric patients with INS, diagnosed during the pandemic (n=29) and post-pandemic (n=30) period, analyzing demographic, clinical, biological and therapeutic variables.

**Results:**

Patient distribution was similar between periods. Hospital stay was longer during the pandemic (median 10, IQR 5 vs. median 8, IQR 4; p = 0.015), whereas the interval from symptom onset to presentation did not differ significantly between periods. Infections associated with onset were less frequent during the pandemic compared to the post-pandemic period (31% vs. 60%; p=0.026), without differences in severity. Corticosteroid-response categories are reported descriptively because follow-up duration differed substantially between the two cohorts, limiting complete ascertainment of corticosteroid dependence in the post-pandemic group. A lower proportion of corticosteroid-sensitive patients was observed during the pandemic, while corticosteroid responsiveness was associated with time to remission (ρ = 0.439; p = 0.003). Exploratory logistic regression suggested that the post-pandemic period, anemia at diagnosis, and weight status were associated with infection-associated onset. In an exploratory logistic regression model, macroscopic hematuria was the only significant clinical variable associated with the observed corticosteroid response (p = 0.039). Biological parameters were similar between periods, except for higher fibrinogen (p = 0.021) and complement C4 (p = 0.034) levels during the pandemic. Exploratory descriptive analyses of the subgroup with available C4 measurements did not identify statistically significant associations between C4 levels and corticosteroid-response categories or other examined clinical characteristics.

**Conclusions:**

Children diagnosed during the pandemic experienced longer hospitalizations, whereas most demographic and biological characteristics were comparable between periods. Infection-associated onset was less frequent during the pandemic than during the post-pandemic period, whereas the number of diagnosed cases was similar between the two study periods. Complement C4 differed between the pandemic and post-pandemic cohorts, but exploratory descriptive analyses did not identify statistically significant associations between C4 levels and corticosteroid response or other examined clinical characteristics. Hematuria was associated with corticosteroid response, whereas anemia and weight status were associated with infection-related onset in exploratory analyses. However, these findings should be interpreted cautiously because of the limited sample size and require confirmation in larger prospective studies.

## 1 Introduction

Nephrotic syndrome in children is one of the most common pediatric glomerular diseases, characterized by massive proteinuria, hypoalbuminemia, generalized edema, and hyperlipidemia. Most cases are idiopathic, with impaired glomerular barrier selectivity and predominant urinary albumin loss. At the cellular level, podocyte dysfunction and alteration, probably immunologically mediated by T-cell and cytokine dysregulation, constitute the central mechanism of proteinuria. This causes a decrease in plasma oncotic pressure, favoring the appearance of edema, and stimulates hepatic synthesis of lipoproteins, explaining the characteristic hyperlipidemia. Although protein loss is generally selective, more severe or secondary forms may be associated with the elimination of other plasma proteins, such as immunoglobulins or coagulation factors, which may increase the risk of infections and hemostatic complications (1–4).

In the pre-pandemic period, the onset and recurrences of nephrotic syndrome were frequently precipitated by common viral infections or nonspecific immuno-inflammatory activations in susceptible children. In the context of the COVID-19 pandemic, SARS-CoV-2 infection has been reported as a triggering factor similar to other viruses, temporally associated with the onset of nephrotic syndrome in children without a history of renal disease. Case reviews and recent reports document over 40 children with onset or relapse of nephrotic syndrome associated with SARS-CoV-2 infection. Most patients had a benign course and responded favorably to standard corticosteroid therapy, reinforcing the hypothesis of a temporal link between viral infection and disease onset, without evidence that the mechanisms are fundamentally different from other infections (5–7).

In addition to the direct effects of infection, the pandemic has led to epidemiological changes, changes in exposure to other infectious agents, and changes in medical practices (e.g., restrictions on access to health services, changes in vaccination prophylaxis, and changes in health behaviors), all of which may influence the frequency, severity, and clinical presentation of nephrotic syndrome during pandemic and post-pandemic periods (8–12). This complex dynamic justifies a comparative analysis between the pandemic and post-pandemic eras, to better understand the impact of SARS-CoV-2 on the onset and evolution of nephrotic syndrome in children and to define optimal diagnostic and management strategies.

## 2 Materials and methods

### 2.1 Study design

We conducted a retrospective observational study conducted at the “Santa Maria” Children’s Emergency Hospital, Iasi - Regional Center for Pediatric Nephrology in North East Moldova (Romania), which targeted pediatric patients newly diagnosed with idiopathic nephrotic syndrome between January 2020 and December 2024. All cases were selected based on medical registries. For comparative analysis, the study period was divided into two intervals: the pandemic period (January 2020 - March 2022) and the post-pandemic period (April 2022 - December 2024). This classification was used to facilitate the statistical evaluation of differences in diagnostic trends, clinical characteristics at onset, disease progression and response to treatment between the two periods.

### 2.2 Study population

The study targeted the pediatric population aged between 1 and 18 years, identified in the hospital database with the diagnosis of idiopathic nephrotic syndrome (summarizing the clinical-biological criteria according to the guidelines in force at that time) during the mentioned time interval. Considering the particularities of the studied period, we chose to include in the research both children who entered the clinic’s records since the onset of the disease, as well as those who presented later, during evolution, for specialized monitoring. Children with non-idiopathic nephrotic syndrome (secondary, congenital, infantile type) or who had a single presentation, after the onset, redirecting to the referral center for continued monitoring were excluded.

### 2.3 Data collection

Relevant demographic, clinical, biological and therapeutic data were retrospectively retrieved from observation sheets, medical records and the center’s database, and processed using dedicated software. Complete remission was defined according to the contemporary pediatric nephrotic syndrome guidelines as negative or trace proteinuria for three consecutive days on urine dipstick (or equivalent documentation in the medical records). Time to remission was calculated from treatment initiation until the first documented complete remission. To minimize extraction errors and ensure data reliability, the information was collected in duplicate by two independent evaluators from the research team and subsequently reviewed by a third member, thereby guaranteeing the consistency and accuracy of the information included in the study. All data were checked for duplicates based on case details.

### 2.4 Statistical analysis

All data were processed and analyzed using Jamovi version 2.6.44. The assessment of normality was based on the combined interpretation of the Shapiro–Wilk test and graphical inspection of histograms and Q–Q plots. Variables were considered approximately normally distributed when the Shapiro–Wilk test did not indicate a significant departure from normality (p > 0.05) and graphical assessment supported this assumption. Variables with an approximately normal distribution were summarized as mean ± standard deviation (SD) and analyzed using parametric tests. Variables that did not meet the normality assumption were summarized as median (interquartile range [IQR]) and analyzed using non-parametric tests. When appropriate, minimum–maximum values or 95% confidence intervals are additionally reported. The choice of parametric or non-parametric tests was based on these assessments. The comparison of groups, pandemic period versus post-pandemic period, was performed using statistical tests appropriate to the type of data and their distribution: parametric tests (t-test for independent samples, Welch t-test) or non-parametric tests (Mann–Whitney U, Chi-square, Fisher exact), as appropriate. For normally distributed continuous variables, homogeneity of variances was assessed using Levene’s test. Student’s independent samples t-test was applied when the assumption of equal variances was met (Levene’s test p > 0.05), whereas Welch’s t-test was used when the assumption of homogeneity of variances was violated (Levene’s test p < 0.05). Categorical variables were compared using the Chi-square test when expected cell counts were adequate. Fisher’s exact test was applied when one or more expected cell counts were <5, owing to the small sample size and sparse contingency tables.

In addition, regression analyses were performed to explore potential predictors of selected clinical and biological outcomes. Binary logistic regression was used for dichotomous outcomes, whereas multiple linear regression was applied for continuous outcomes. For multiple linear regression models, the overall model significance was assessed using analysis of variance (ANOVA), and regression coefficients (β), coefficients of determination (R² and adjusted R²), root mean square error (RMSE), and corresponding p-values were reported. Odds ratios (OR) with 95% confidence intervals were reported for logistic regression models. Model assumptions were evaluated by examining residual normality (Shapiro–Wilk test and Q–Q plots), multicollinearity (variance inflation factors), influential observations (Cook’s distance), and multivariate outliers (Mahalanobis distance). Time to remission was analyzed using Kaplan–Meier survival curves and Cox proportional hazards regression, with hazard ratios (HRs) and 95% confidence intervals reported after verification of the proportional hazards assumption. Associations between continuous or ordinal variables were assessed using Spearman’s rank correlation coefficient when appropriate. The apparent in-sample discriminatory performance of logistic regression models was evaluated using receiver operating characteristic (ROC) curve analysis, including the area under the curve (AUC), sensitivity, and specificity at a probability threshold of 0.5. Owing to the exploratory nature of these analyses and the limited sample size, no internal validation procedures were performed. Effect sizes were reported where appropriate, including Cohen’s d, rank-biserial correlation, Phi coefficient, Cramér’s V, odds ratios, and hazard ratios. For all statistical tests, statistical significance was defined as p < 0.05, and 95% confidence intervals were reported where applicable.

### 2.5 Ethical approval

The study was conducted in accordance with the principles of the Declaration of Helsinki and is part of the research carried out within the doctoral program. The protocol was approved by the Ethics Committee of the University of Medicine and Pharmacy “Grigore T. Popa” Iași (499/30.11.2024), as well as by the Ethics Committee of the “Santa Maria” Children’s Emergency Hospital Iași (38793/20.11.2024). The data were collected retrospectively from the medical records and processed anonymously, respecting all legal and ethical provisions regarding the confidentiality and protection of patient information.

## 3 Result

The main outcome of the data analysis aims to identify any differences between the pandemic and post-pandemic periods, providing a detailed picture of the patient profile and dynamics of idiopathic nephrotic syndrome within the studied cohort. Secondary outcomes included descriptive analysis of patient characteristics (demographic, clinical and paraclinical) and statistical correlations between disease onset, symptom severity, response to therapy and mid-term evolution.

### 3.1 General characteristics of the cohort

After consulting the electronic registers, excluding patients already registered or known to have had their onset before the mentioned date, as well as those diagnosed with congenital nephrotic syndrome (3 patients), infantile nephrotic syndrome (1 patient) and cases in which the referral to our center was through a single presentation, subsequently returning to the initial reference center (1 patient), a total of 59 patients were enrolled in the study. Overall, the cohort is characterized by a male predominance, relatively early onset of the disease and the predominance of corticosteroid-sensitive forms, although a relevant proportion of patients presents a more difficult therapeutic evolution. The general characteristics of the cohort are summarized in **Table 1**. Statistical analysis of the data showed a numerically higher proportion of boys (35, 59.3%) than girls (24, 40.7%). Age at onset was expressed in months, with a median of 43 months (IQR 57), ranging from 13 to 207 months across the entire cohort. Gender-based analysis revealed a trend towards earlier onset in boys (median 36 months; IQR 45) compared to girls (median 48.5 months; IQR 65,8). Regarding the environment of origin, 21 patients (35.6%) came from an urban environment, the distribution being relatively similar between genders. In terms of response to corticosteroid therapy, most patients were corticosteroid-sensitive (34 cases, 57.63%), while 11 patients (18.64%) showed corticosteroid dependence, and 14 patients (23.73%) were corticosteroid-resistant. The gender distribution indicates a higher proportion of corticosteroid-sensitive patients among boys (64.71%) compared to girls (35.29%), while the frequency of dependence and resistance to corticosteroid therapy was relatively comparable between the two groups.

**Table 1:** Demographic characteristics of the cohort.

Characteristic	Total n (%)	Boys	Girls	p - value	Test
Number (%)	59 (100%)	35 (59.32%)	24 (40.68%)	–	–
Median age (IQR)	43 months (57)	36 months (45)	48,5 months (65,8)	0,277	Mann-Whitney
Environment				0,420	Chi-square
Urban	21 (35.59%)	11 (52,38%)	10 (47,62%)		
Rural	38 (64,41%)	24 (63,16%)	14 (36,84%)		
Corticoresponsivity				0,519	Chi-square
Corticoresensitive (%)	34 (57,63%)	22 (64,71%)	12 (35,29%)		
Corticoredependent (%)	11 (18,64%)	5 (45,45%)	6 (54,54%)		
Corticoreresistant (%)	14 (23,73%)	8 (57,14%)	6 (42,86%)		

### 3.2 Demographic characteristics and evolution in pandemic versus post-pandemic context

Overall, the pandemic and post-pandemic cohorts were demographically comparable, whereas the main differences were related to healthcare utilization, including longer hospitalization and greater variability in remission during the pandemic period. The analysis of the distribution of patients according to the period of diagnosis (**Table 2**) revealed an almost equal distribution between the pandemic (29 patients, 49.15%) and post-pandemic (30 patients, 50.85%) period, which indicates a comparable distribution of diagnosed cases between the two study periods within our cohort. The distribution by gender was comparable between periods: in the pandemic 15 boys (51.72%) and 14 girls (48.28%) versus 20 boys (66.67%) and 10 girls (33.33%) post-pandemic (χ² = 1.36, df = 1, Phi = 0.152, p = 0.243). The environment of origin did not reveal significant differences (urban 11/29, 37.93% versus 10/30, 33.33%, χ² = 0.136, df = 1, Phi = 0.048, p = 0.712). These observations indicate that the two cohorts were comparable with respect to gender distribution and demographic characteristics, supporting the validity of the comparison between study periods.

**Table 2:** Comparative epidemiological distribution of patients in the pandemic and post-pandemic periods.

Variable	Subcategory / group	Pandemic period (N)	Statistics / % / median, IQR; min-max	Post-pandemic period (N)	Statistics / % / median, IQR; min-max)	P value / effect	Test
Total number of patients	–	29	49,15%	30	50,85%		
Gender	Boys	15	51,72%	20	66,67%	p = 0,243	Chi-square
	Girls	14	48,28%	10	33,33%	–	
Environment of origin	Rural	18	62,07%	20	66,67%	p = 0,712	Chi-square
	Urban	11	37,93%	10	33,33%	–	
Mode of presentation	Direct onset	20	68.97% (10 direct, 10 transfer)	28	93,33% (13 direct, 15 transfer)	p = 0,054; Cramér’s V = 0,314	Chi-square
	Later acute episode	9	31,03%	2	6,67%	–	
Time to presentation (days)	–	15	Median 7, IQR 11.5; min–max 1–90	25	Median 4, IQR 8; min–max 1–30	U = 167, p = 0,573; r = 0,109;	Mann–Whitney
						W = 0,511, p = 0,001; W = 0,736, p = 0,001	Shapiro–Wilk: • pandemic • post-pandemic
Duration of hospital stay (days)	–	21	Median 10, IQR 5, min–max 5–23	28	Median 8, IQR 4, min–max 4–21	U = 174, p = 0,015; r = 0,410;	Mann–Whitney
						W = 0,903, p = 0,04; W = 0,848, p < 0,001	Shapiro–Wilk: • pandemic • post-pandemic
Time to remission (days)	–	22	Median 40, IQR 13.5, min–max 6–1569	23	Median 36, IQR 15, min–max 5–352	U = 191, p = 0,159; r = 0,247;	Mann–Whitney
						W = 0,567, p = 0,001; W = 0,298, p = 0,001	Shapiro–Wilk: • pandemic • post-pandemic
						40 (IC95%: 38–49); 36 (IC95%: 31–46),	Kaplan–Meier estimator • pandemic • post-pandemic
						Cox proportional hazards model: HR = 0,75 (IC95%: 0,41–1,36), p = 0,341. Concordance = 0.564. Proportional hazards assumption: χ² = 1.475, p = 0.2246.	
Corticosensitivity	Sensitive	13	44,83%	21	70%	–	
	Dependent	7	24,14%	4	13,3%	–	
	Resistant	9	31,03%	5	16,7%	–	
Correlation corticosensitivity – remission duration	–	–	Spearman ρ = 0,439, p = 0,003	–	–	Moderate, statistically significant association	
Age at onset (months)	–	29	Median 43, IQR 60, min–max 14–207 months	30	Median 42, IQR 51, min–max 13–136 months	U = 424, p = 0,868; r = 0,026;	Mann–Whitney
						W = 0,769, p = 0,001; W = 0,879, p = 0,003	Shapiro–Wilk: • pandemic • post-pandemic
Mean follow-up period (months)	–	29	Median 35, IQR 35, min–max. 5–60	30	Mediană 6.5, IQR 9,75, min–max 0–32	–	

Regarding the mode of presentation, 20 patients (68.97%) from the pandemic period presented at the time of onset (10 directly in our health unit, 10 by transfer/redirection from the territory), and 9 patients (31.03%) were taken after the acute episode, compared to 28 patients (93.33%) post-pandemic (13 directly in our health unit, 15 by transfer/redirection from the territory) and only 2 patients (6.67%) after the acute episode (χ² = 5.83, df = 2, Cramér’s V = 0.314, p = 0.054). Although the difference did not reach the threshold of significance, the associated magnitude indicates a moderate effect. This trend is also reflected in the time elapsed between symptom onset and presentation to a medical service: a median of 7 days (IQR 11.5, min–max 1–90) during the pandemic versus a median of 4 days (IQR 8, min–max 1–30) post-pandemic (Mann–Whitney U = 167, rank-biserial r = 0.109, p = 0.573). Extreme values indicate that a small number of patients experienced prolonged intervals between symptom onset and presentation. Although the overall difference between groups was not statistically significant, such individual delays may have clinical relevance.

The average length of hospital stay was significantly longer during the pandemic period (median 10, IQR 5, min–max 5–23) compared to the post-pandemic period (median 8, IQR 4, min–max 4–21) (Mann–Whitney U = 174, rank-biserial r = 0.410, p = 0.015). This difference may reflect changes in healthcare organization, including isolation procedures and monitoring protocols during the pandemic period.

The time to remission showed marked variability, particularly during the pandemic period (median 40 days, IQR 13.5, min–max 6–1569) compared to the post-pandemic period (median 36 days, IQR 15, min–max 5–352). This difference was not statistically significant (Mann–Whitney U = 191, rank-biserial r = 0.247, p = 0.159); however, the extreme variability observed during the pandemic reflects the presence of cases with a prolonged clinical course. The reasons for this variability cannot be determined from the present data. Survival analysis using the Kaplan–Meier estimator revealed a median time to remission of 36 days (95% CI: 31–46) in the post-pandemic period and 40 days (95% CI: 38–49) in the pandemic period, with no significant differences between the groups. In the Cox proportional hazards regression model, the pandemic period was associated with a slightly lower hazard of remission compared to the post-pandemic period (Cox proportional hazards regression: HR = 0.75, 95% CI: 0.41–1.36, p = 0.341), though this association was not statistically significant. The proportional hazards assumption was met, supporting the suitability of the model used. The longer median follow-up period during the pandemic (35 months, IQR 35, range 5–60 months) versus the post-pandemic period (6.5 months, IQR 9.75, range 0–32 months) partly explains the increased dispersion and clinical relevance of the observations, providing a comprehensive perspective on disease progression and treatment response.

Overall corticosteroid-response categories are presented descriptively because follow-up duration differed substantially between the two cohorts, particularly for corticosteroid dependence, which requires prolonged observation. During follow-up, 13/29 (44.8%) patients in the pandemic cohort and 21/30 (70.0%) in the post-pandemic cohort were classified as corticosteroid-sensitive, whereas corticosteroid dependence was observed in 7/29 (24.1%) and 4/30 (13.3%) patients, respectively, and corticosteroid resistance in 9/29 (31.0%) and 5/30 (16.7%). Spearman correlation demonstrated a moderate and significant association between the degree of corticosensitivity and the duration to remission (ρ = 0.439, p = 0.003). This result suggests that patients with a reduced therapeutic response require longer periods of treatment and monitoring.

Age at onset was comparable between the two periods: the median was 43 months (IQR = 60; min–max: 14–207 months) during the pandemic period and 42 months (IQR = 51; min–max: 13–136 months) during the post-pandemic period. Mann–Whitney U test analysis revealed no statistically significant differences between the groups and the effect size was negligible (U = 424, rank-biserial r = 0.026, p = 0.868). These findings indicate that age at onset was comparable between the two periods and is therefore unlikely to account for the observed differences between the cohorts.

### 3.3 Clinical manifestations and biological profile of patients

The main clinical difference between the two periods was the higher frequency of infection-associated onset in the post-pandemic period, whereas most other clinical and laboratory parameters remained comparable (**Table 3**). During the pandemic period, only 9 patients (31.0%) presented infections correlated with the onset, compared to 18 patients (60.0%) in the post-pandemic period. This difference was statistically significant (χ² = 4.98, df = 1, Phi = 0.291, p = 0.026), the effect estimate indicating that patients in the post-pandemic period were 3.33 times more likely to associate the onset of nephrotic syndrome with an infectious episode (95% CI: 1.14–9.75), and the Phi = 0.291 reflects a moderate association between the period and the occurrence of infections. We note that, both during the pandemic and post-pandemic periods, no patient reported acute infection with SARS Cov-2 virus correlated with the time of onset.

**Table 3:** Onset of nephrotic syndrome and associated clinic biological factors between pandemic and post-pandemic periods.

Variable / predictor	Pandemic (N = 29)	Post-pandemic (N = 30)	Test	P-value	Effect size / OR (95% CI)
Infection associated with the onset	9 (31.0%)	18 (60.0%)	Chi-square	0.026	OR = 3.33 (1.14–9.75)
Severity of infection			Fisher’s Exact Test	p = 0.692	OR = 1.60 (0.30–8.49)
Mild	3 (33.3%)	8 (44.4%)			
Moderate	6 (66.7%)	10 (55.6%)			
Gross hematuria	3 (10.3%)	2 (6.7%)	Fisher’s Exact Test	0.671	OR = 0.619 (0.10-4.01)
Anemia at diagnosis	4 (13.8%)	4 (13.3%)	Fisher’s Exact Test	1.000	OR = 0.962 (0.217-4.27)
Increased blood pressure values	2 (6.9%)	4 (13.3%)	Fisher’s Exact Test	0.671	OR = 2.08 (0.350-12.3)
Initial therapeutic need			Mann–Whitney U = 303	0.215	Rank-biserial = −0.166
Albumin	2 (6.9%)	5 (16.7%)			
Albumin + furosemide	4 (13.8%)	7 (23.3%)			
No albumin required	23 (79.3%)	18 (60.0%)			
BMI after edema remission			Mann–Whitney U = 390	0.577	Rank-biserial = 0.073
Underweight	0 (0%)	1 (3.3%)			
Normal weight	18 (62.1%)	20 (66.7%)			
Overweight	11 (37.9%)	9 (30.0%)			
Predictors – Logistic regression					
Post-pandemic period	—	—	Likelihood Ratio Test	0.010	OR = 4.99 (1.36–18.24)
Anemia at diagnosis	—	—	Likelihood Ratio Test	0.012	OR = 11.67 (1.11–122.44)
BMI category	—	—	Likelihood Ratio Test	0.039	OR = 0.24 (0.064–0.901)
Macroscopic hematuria	—	—	Likelihood Ratio Test	0.932	—
Elevated blood pressure	—	—	Likelihood Ratio Test	0.921	—
Logistic model performance	—	—	—	—	AUC = 0.808; Sensitivity = 93.5%; Specificity = 55.6%; Cut-off = 0.5

The severity of infections at onset was analyzed, being defined as: mild infections – effectively treated symptomatically, moderate infections – requiring specific pathogen treatment, severe infections – accompanied by evolving complications, requiring intensive care. During the pandemic period, most infections were moderate (6 patients; 66.7%), while 3 patients (33.3%) had mild infections. In the post-pandemic period, the distribution of severity was more balanced: 8 patients (44.4%) mild and 10 patients (55.6%) moderate. No patients presented with severe infections at disease onset in either study period. Comparison of infection severity between the pandemic and post-pandemic periods using Fisher’s exact test showed no significant association (OR = 1.60, 95% CI: 0.30–8.49, p = 0.692).

To further explore factors associated with infection-related onset, an exploratory binary logistic regression analysis was performed (**Table 3**), including macroscopic hematuria, anemia at diagnosis, elevated blood pressure values, weight status (BMI) after edema resolution, and pandemic versus post-pandemic period as candidate predictors. The corresponding Omnibus likelihood ratio tests (Omnibus LR test) supported the contribution of these variables to model fit:

- The post-pandemic period remained a statistically significant predictor of the onset-infection correlation, (Omnibus LR test: χ² = 6.62, df = 1, p = 0.010), suggesting that patients in the post-pandemic period had higher odds of presenting with nephrotic syndrome associated with an infectious episode.
- Anemia at diagnosis was also a statistically significant predictor (Omnibus LR test: χ² = 6.30, df = 1, p = 0.012), suggesting a possible association between anemia at diagnosis and infection-related onset.
- Weight status in remission (BMI) had a significant effect on the probability of onset associated with infection (Omnibus LR test: χ² = 6.48, df = 2, p = 0.039). This finding suggests a possible inverse association between overweight status and infection-related onset.
- Macroscopic hematuria and elevated blood pressure values at diagnosis were not statistically significant predictors (Omnibus LR test: χ² = 0.007, df = 1, p = 0.932, respectively χ² = 0.010, df = 1, p = 0.921, indicating that they were not significantly associated with infection-related onset in this cohort.

The ROC curve of the exploratory model is presented in **Supplementary Figure 1**. Because discrimination was assessed using the same dataset on which the model was developed and no internal or external validation was performed, the observed AUC (0.808) represents only apparent model discrimination and should not be interpreted as evidence of clinical predictive performance. Overall, these findings should be regarded as exploratory and hypothesis-generating, requiring confirmation in larger independent cohorts.

Because of the low frequencies observed for macroscopic hematuria (n = 5), anemia at diagnosis (n = 8), and elevated blood pressure (n = 6), Fisher’s exact test was used for these comparisons. Analysis of other clinical parameters at the time of diagnosis did not reveal significant differences between periods. Macroscopic hematuria was reported in 5 patients (8.5%), with similar distribution between periods (Fisher’s exact test, OR = 0.619, 95% CI: 0.10-4.01, p = 0.671). Anemia at diagnosis was observed in 4 patients in each group (Fisher’s exact test, OR = 0.962, 95% CI: 0.217-4.27, p = 1.000). Elevated blood pressure values were identified in 6 patients (10.2%), with no significant differences between periods (Fisher’s exact test, OR = 2.08, 95% CI: 0.350-12.3, p = 0.671).

The assessment of the initial therapeutic need, by administering albumin or albumin associated with diuretic, did not reveal significant differences between periods (Mann–Whitney U = 303, rank-biserial r = −0.166, p = 0.215). Regarding nutritional status, assessed by body mass index (BMI) after edema resolution, the distribution of weight categories did not show significant variations between groups (Mann–Whitney U = 390, rank-biserial r = 0.073, p = 0.577), indicating a negligible association between the period of diagnosis and weight status after edema resolution.

**Figure 1:**
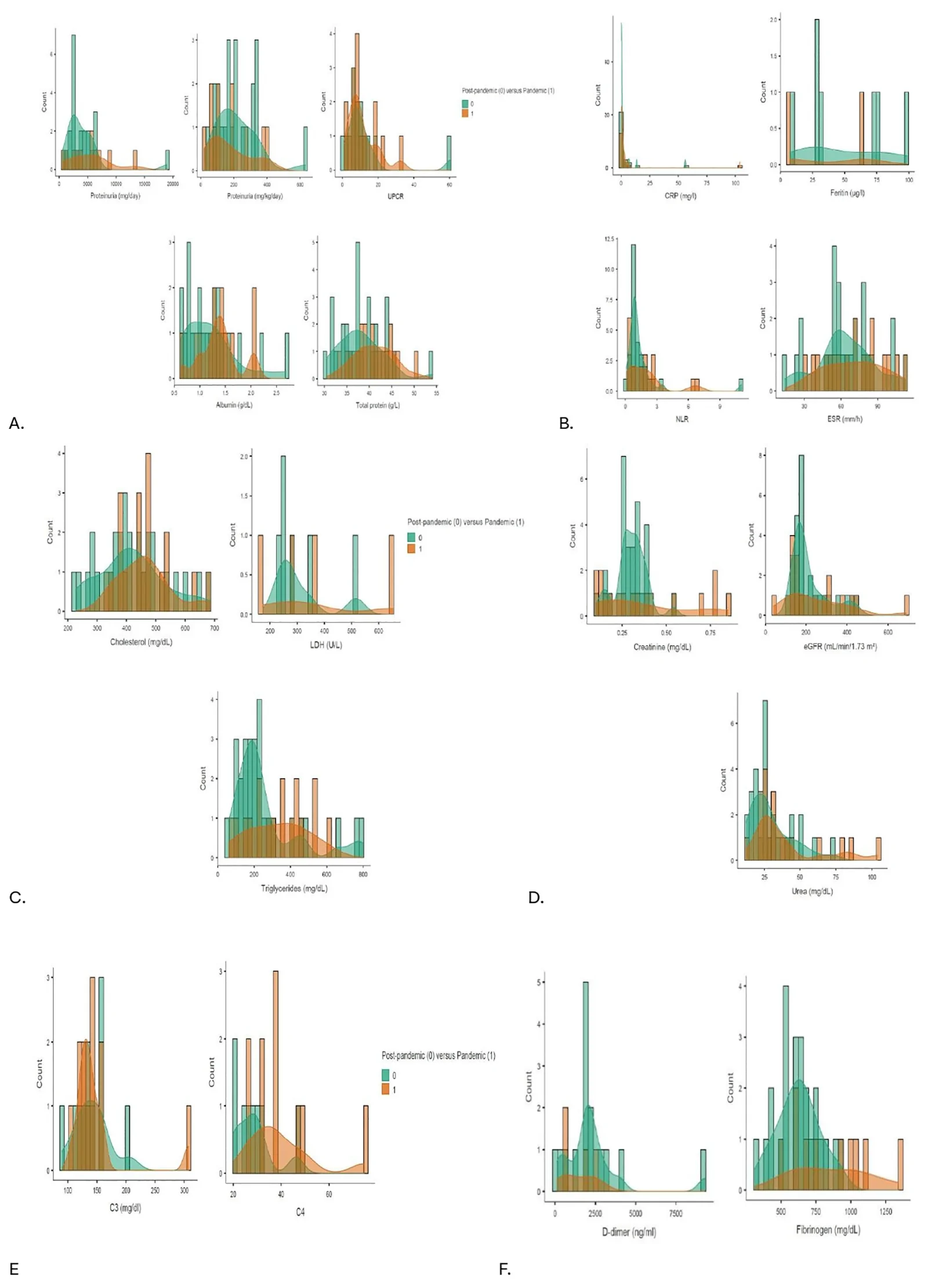
Distribution of laboratory parameters in the post-pandemic and pandemic periods. Histograms with density curves illustrate the distribution of proteinuria-related and serum protein parameters **(A)**, inflammatory and hematological parameters **(B)**, lipid and metabolic parameters **(C)**, renal function parameters **(D)**, complement components **(E)**, and coagulation markers **(F)** in the post-pandemic period (0) and pandemic period (1). Specifically, panel A shows proteinuria, proteinuria/creatinine ratio, UPCR, albumin, and total protein; panel B shows CRP, ferritin, NLR, and ESR; panel C shows cholesterol, LDH, and triglycerides; panel D shows creatinine, eGFR, and urea; panel E shows C3 and C4; and panel F shows D-dimer and fibrinogen. Green and orange distributions represent the post-pandemic and pandemic periods, respectively. UPCR, urine protein-to-creatinine ratio; CRP, C-reactive protein; NLR, neutrophil-to-lymphocyte ratio; ESR, erythrocyte sedimentation rate; eGFR, estimated glomerular filtration rate; LDH, lactate dehydrogenase.

**Figure 2:**
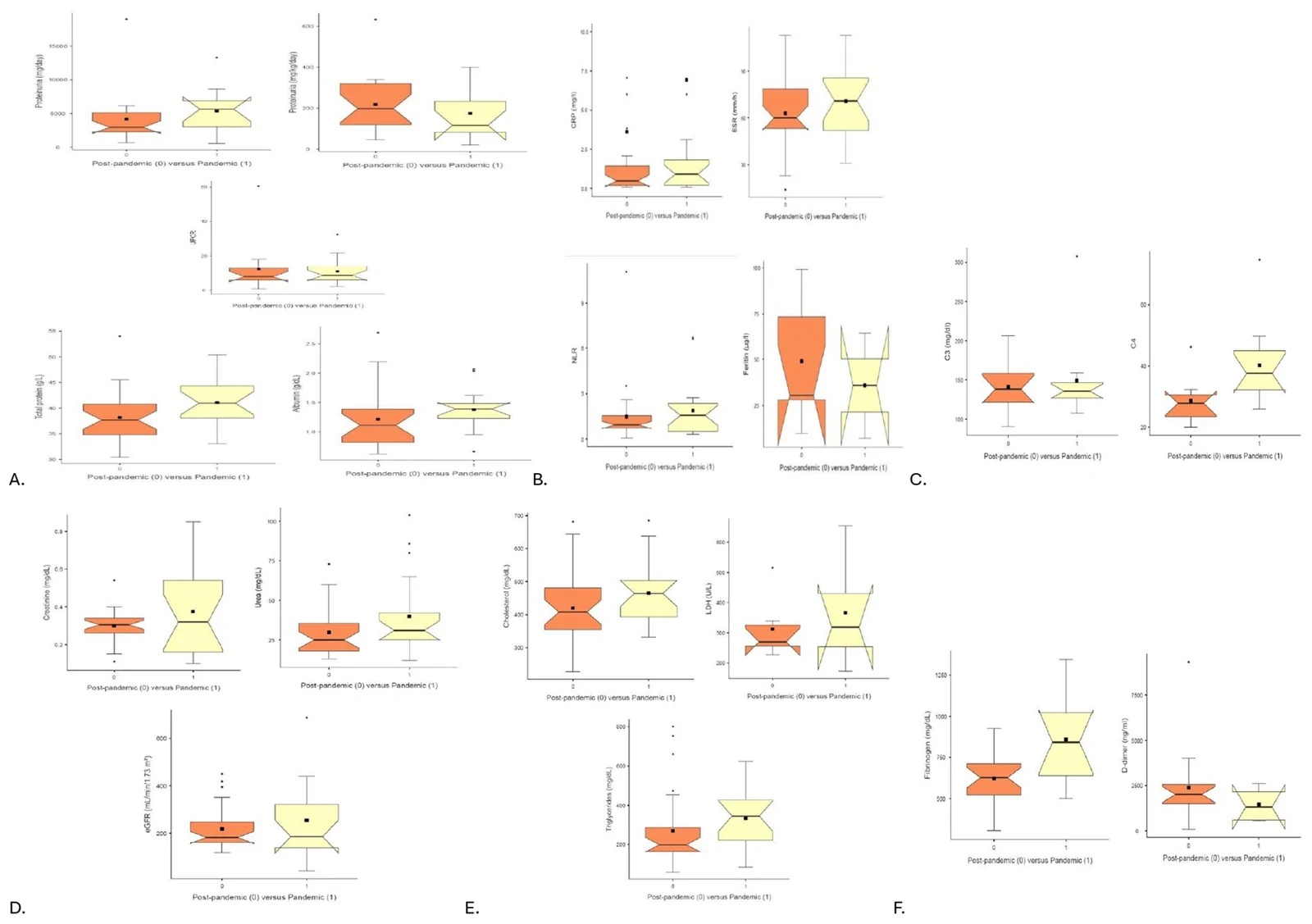
Comparison of laboratory parameters between the post-pandemic and pandemic periods. Box-and-whisker plots show the distribution of proteinuria-related and serum protein parameters **(A)**, inflammatory and hematological parameters **(B)**, complement components **(C)**, renal function parameters **(D)**, lipid and metabolic parameters **(E)**, and coagulation markers **(F)** in the post-pandemic period (0) versus the pandemic period (1). Specifically, panel A shows proteinuria, proteinuria/creatinine ratio, UPCR, albumin, and total protein; panel B shows CRP, ESR, NLR, and ferritin; panel C shows C3 and C4; panel D shows creatinine, urea, and eGFR; panel E shows cholesterol, LDH, and triglycerides; and panel F shows fibrinogen and D-dimer. The boxes represent the interquartile range, the horizontal lines indicate the median, black squares indicate the mean, whiskers represent the range, and individual points indicate outliers. UPCR, urine protein-to-creatinine ratio; CRP, C-reactive protein; ESR, erythrocyte sedimentation rate; NLR, neutrophil-to-lymphocyte ratio; eGFR, estimated glomerular filtration rate; LDH, lactate dehydrogenase.

Furthermore, to compare the clinical and biological presentation of nephrotic syndrome between the pandemic and post-pandemic periods, we analyzed a comprehensive set of urinary, serum, inflammatory and coagulation parameters (**Table 4**). The distribution of the values of each parameter was represented by a histogram (**Figure 1**), and the statistical comparison between groups was performed by a box plot (**Figure 2**), which highlights the medians, interquartile ranges and extreme values. Both figures are structured by categories of biological markers: protein balance, metabolic, inflammatory, renal function, complement and coagulation biomarkers. The analysis aimed to identify significant differences that could reflect both changes in nephrotic severity and differential immunological and inflammatory responses.

**Table 4:** Biological parameters at the onset of nephrotic syndrome in children: comparison between the pandemic and post-pandemic period.

Parameter	Post-pandemic (0) mean ± SD / median (IQR), min-max	Pandemic (1) mean ± SD / median (IQR), min-max	Shapiro-Wilk	Mann–Whitney U / T test/ Welch`s test
Proteinuria (mg/day)	2968 (2825), 677–19000	5659 (3882), 568–13292	W=0.642 p<0.001 (0); W = 0.950 p=0.640 (1)	U=89.0, p=0.176, rb=0.2964
Proteinuria (mg/kgc/day)	198 (202), 45.1–633	116 (152), 20–400	W=0.872 p=0.011 (0); W = 0.892 p=0.148 (1)	U=88.5, p=0.293, rb=-0.2338
Protein/creatinine ratio	7.93 (7.04), 0.756–60.5	8.66 (7,86), 2.26–32.4	W=0.612 p<0.001 (0); W = 0.869 p=0.027 (1)	U=98.0, p=0.812, rb=0.0577
Serum protein (g/dl)	38.2 ± 5.18	41.1 ± 4.31	W=0.941 p=0.118 (0); W = 0.989 p=0.997 (1)	t(45) = −2.01, p = 0.051, Cohen’s d = −0.597
Albuminemia (g/dl)	1.11 (0.569), 0,620-2.69	1.39 (0.253), 0.663-2.07	W=0.882 p=0.016 (0); W = 0.954 p=0.658 (1)	U=95.0, p=0.146, rb=0.3040
Cholesterol (mg/dl)	420 ± 117	465 ± 92.2	W=0.973 p=0.667 (0); W = 0.937 p=0.228 (1)	t(45) = −1.41, p = 0.164, Cohen’s d = −0.421
Triglycerides (mg/dl)	199 (124), 60–800	345 (207), 84–623	W=0.789 p<0.001 (0); W = 0.971 p=0.830 (1)	U=172.0, p=0.125, rb=0.2773
LDH (U/L)	271 (70.5), 227–516	319 (177), 173–654	W=0.794 p<0.001 (0); W = 0.924 p=0.559 (1)	U=10.0, p=0.762, rb=0.1667
C3 (mg/dl)	138 (36.7) 90-206	136 (19.8) 107-308	W=0.962 p=0.802 (0); W = 0.598 p<0.001 (1)	U=64.0, p=0.926, rb=-0.0303
C4 (mg/dl)	27.8 (7.16) 20-46.2	37.5 (12.7) 25.9-74.7	W=0.880 p=0.188 (0); W = 0.843 p=0.048 (1)	U=16.0, p=0.034, rb=0.6000
Creatinine (mg/dl)	0.305 (0.0800) 0.110-0.540	0.320 (0.380) 0.100-0.850	W=0.950 p=0.203 (0); W = 0.867 p=0.020 (1)	U=235.0, p=0.953, rb=0.0126
Urea (mg/dl)	25 (17.5) 13-73	31 (17) 12-104	W=0.874 p=0.003 (0); W = 0.796 p<0.001 (1)	U=195.0, p=0.125, rb=0.2669
GFR (mL/min/1.73 m²)	182 (86.4) 120-450	186 (182) 40.8-688	W=0.812 p<0.001 (0); W = 0.888 p=0.043 (1)	U=235.5, p=0.963, rb=0.0105
ESR (mm/h)	63.0 ± 24	70.7 ± 24.2	W=0.975 p=0.745 (0); W = 0.975 p=0.885 (1)	t(42) = −1.03, p = 0.307, Cohen’s d = −0.317
Fibrinogen (mg/dl)	622 ± 154	862 ± 267	W=0.991 p=0.997 (0); W = 0.956 p=0.736 (1)	Welch’s t = −2.66, df = 11.6, p = 0.021, Cohen’s d = −1.10
CRP (mg/l)	0.500 (1.25), 0.1–56.1	0.920 (1.59), 0.1–104	W=0.345 p<0.001 (0); W = 0.297 p<0.001 (1)	U=235.5, p=0.871, rb=0.0309
NLR	0.930 (0.810), 0.07–11.1	1.56 (1.84), 0.31–6.73	W=0.487 p<0.001 (0); W = 0.748 p<0.001 (1)	U=235.0, p=0.508, rb=0.1165
Ferritin (μg/l)	30.5 (45.4), 10–98.9	35.9 (28.7), 7.13–64.6	W=0.894 p= 0.294 (0); W=NaN p=NaN (1)	U=4.0, p=0.500, rb=-0.4286
D-dimers (ng/ml)	2002 (1056), 100–9300	1317 (1552), 560–2614	W=0.735 p<0.001 (0); W = 0.858 p=0.255 (1)	U=24.0, p=0.596, rb=-0.2000

Thus, we observe that daily proteinuria was numerically higher in children with onset during the pandemic period (median 5659 mg/day, IQR 3882) compared to those with post-pandemic onset (median 2968 mg/day, IQR 2825), but the difference did not reach statistical significance (Mann–Whitney U = 89.0, rank-biserial r = 0.296, p = 0.176). Similarly, the protein/creatinine ratio and proteinuria adjusted for body weight did not show significant differences between groups. These data suggest that, despite individual variations, the degree of glomerular involvement at onset remained comparable between periods.

Serum total protein and albumin levels showed a trend towards lower values post-pandemic (serum protein: mean 38.2 ± 5.18 g/L vs. 41.1 ± 4.31 g/L; albumin: median 1.11, IQR 0.569 vs. median 1.39, IQR 0.253), suggesting a slightly more pronounced impact of urinary protein losses on systemic protein balance. Lipid profile showed higher triglyceride and cholesterol values during the pandemic period, which may reflect a more pronounced metabolic reaction associated with inflammation and systemic stress, even though these differences were not statistically significant. Renal function, assessed by creatinine, urea and glomerular filtration rate, was comparable between periods. D-dimer values varied between groups, but did not show significant differences. Biomarkers such as CRP, ESR, NLR or ferritin remained comparable, suggesting impairment of the coagulation cascade rather than traditional inflammatory markers.

In our analysis, a significant difference in C4 and fibrinogen levels was observed between the two periods, whereas the remaining biological parameters were comparable. Median C4 values were higher during the pandemic (37.5, IQR 12.7) than during the post-pandemic period (27.8, IQR 7.16). This difference was statistically significant (Mann–Whitney U = 16.0, rank-biserial r = 0.600, p = 0.034) and suggests a transient immunological hyperresponsiveness at onset, although values remained within or only slightly above the normal range, without indicating pathological activation. C3 levels did not show relevant differences between periods. Serum fibrinogen levels were significantly higher during the pandemic period (mean 862 ± 267 mg/dL) than during the post-pandemic period (mean 622 ± 154 mg/dL; Welch’s t = −2.66, df = 11.6, Cohen’s d = −1.10, p = 0.021), indicating a more pronounced procoagulant activation. Other biomarkers, such as CRP, ESR, NLR and ferritin, did not show significant differences.

To further explore variables associated with baseline ESR, an exploratory multivariable linear regression model (**Supplementary Table 1**) including CRP, NLR, fibrinogen, and albumin (N = 25) was constructed. CRP and NLR remained significantly associated with ESR after adjustment for the remaining variables, whereas fibrinogen and albumin showed only marginal associations. Given the limited sample size and the number of predictors included, these findings should be interpreted as exploratory and hypothesis-generating. Detailed regression coefficients, model diagnostics and diagnostic plots are provided in **Supplementary Table 1** and **Supplementary Figures 2**, **3**.

### 3.4 Exploratory analyses of factors associated with corticosteroid response

An exploratory multivariable logistic regression model was fitted to examine variables associated with corticosteroid response. The dependent variable was the evolution under corticosteroid therapy, coded binary: 0 = corticosteroid-sensitive, 1 = initially non-corticosteroid-sensitive (defined as corticosteroid-dependent or corticosteroid-resistant), and the included predictors were macroscopic hematuria at onset, albuminemia at diagnosis, duration until remission, patient gender, correlation of disease onset with infections and presence of anemia. Gross hematuria was the only variable significantly associated with non-corticosteroid-sensitive disease within this exploratory model. Detailed regression coefficients, model diagnostics and the apparent ROC curve derived from the development dataset are provided in the Supplementary Material (**Supplementary Table 2**; **Supplementary Figure 4**). Because of the limited sample size, multiple predictors and the absence of internal or external validation, the model should be regarded as exploratory and hypothesis-generating rather than clinically predictive.

The logit regression coefficients revealed that the only statistically significant predictor for non-corticoid sensitivity was gross hematuria, suggesting an association between baseline gross hematuria and non-corticosteroid-sensitive disease within this exploratory model. The coefficient for albuminemia at diagnosis indicated a trend consistent with clinical expectations: lower serum albumin levels were associated with a higher risk of non-corticoid sensitivity, although the effect did not reach statistical significance. Additional predictors—time to remission, patient gender, correlation of onset with infections, and presence of anemia—had small and statistically insignificant effects (p > 0.1) but were retained in the model to allow for clinical adjustment of the analysis. The analysis suggests that a longer time to remission is associated with an increased likelihood of corticosteroid insensitivity. However, the distribution of the data (**Supplementary Figure 5**) shows that long times are underrepresented, leading to unstable estimates and wide confidence intervals in this area. Because of the limited sample size and lack of external validation, the model should be considered exploratory and hypothesis-generating rather than suitable for clinical prediction.

Finally, because C4 was the only complement component that differed significantly between the pandemic and post-pandemic cohorts, we performed exploratory descriptive analyses to evaluate its relationship with selected clinical characteristics. Owing to the small number of patients with available C4 measurements (n = 18), only univariable comparisons were performed. C4 values tended to be higher among patients with non-corticosteroid-sensitive disease (corticosteroid-dependent or corticosteroid-resistant) than among corticosteroid-sensitive patients (median 38.5, IQR 11.5 vs. 29.0, IQR 6.22), although this difference did not reach statistical significance (Mann–Whitney U = 20.0, p = 0.104; rank-biserial correlation = 0.481). Because C4 values were not normally distributed (Shapiro–Wilk W = 0.809, p = 0.002), non-parametric methods were used. Patients with macroscopic hematuria showed numerically higher C4 values than those without hematuria (median 38.5, IQR 14.1 vs. 30.0, IQR 10.6), but the difference was not statistically significant (Mann–Whitney U = 23.0, p = 0.387; rank-biserial correlation = −0.292). Likewise, no significant difference in C4 levels was observed according to the presence of elevated blood pressure at diagnosis (median 32.2, IQR 4.63 vs. 31.3, IQR 12.4; Mann–Whitney U = 24.0, p = 0.721; rank-biserial correlation = −0.143). Age at diagnosis was not correlated with C4 concentration (Spearman’s ρ = −0.092, p = 0.717). Overall, these exploratory univariable analyses did not identify statistically significant associations between C4 levels and corticosteroid response or the examined clinical characteristics. Given the limited sample size, these findings should be interpreted cautiously and considered hypothesis-generating.

## 4 Discussions and limitations

The present study provides a comprehensive analysis of the impact of the pandemic context on the clinical, biological and evolutionary profile of idiopathic nephrotic syndrome in children, highlighting both elements of continuity with classical data from the literature, as well as emerging particularities relevant from a clinical and epidemiological point of view.

Our results reiterate that the demographic characteristics of the diagnosed cases were similar between the two study periods. The male predominance, young age at onset, and the majority of corticosteroid-sensitive patients are consistent with data reported in international cohorts (13–15). Also, the lack of significant differences between periods in terms of proteinuria, renal function and most inflammatory markers suggests that the fundamental pathophysiological mechanisms of the disease were not directly influenced by the COVID-19 pandemic. The wide range of estimated glomerular filtration rate values observed at presentation likely reflects the heterogeneous hemodynamic changes accompanying nephrotic syndrome. Reduced values may result from transient intravascular volume depletion or acute kidney injury, whereas very high estimates may be attributable to the low serum creatinine concentrations commonly observed in children, particularly in younger patients, rather than true pathological hyperfiltration. Therefore, measurements obtained at disease onset should be interpreted cautiously within the overall clinical context. These results are in agreement with those previously reported in the literature (16, 17). Thus, our results suggest that pediatric idiopathic nephrotic syndrome is predominantly determined by intrinsic immunological factors, relatively independent of external epidemiological variations. No major differences in the initial biological severity of disease were observed between the pandemic and post-pandemic cohorts. The main differences identified in this study were related to healthcare utilization and clinical management rather than to the biological presentation at diagnosis.

In contrast to the stability of the biological profile, the results highlight a significant impact of the pandemic on the clinical course of patients. Although the median interval between symptom onset and presentation was longer during the pandemic, this difference did not reach statistical significance. The longer hospitalization observed during the pandemic may reflect multiple factors, including changes in healthcare organization, infection-control protocols, or differences in clinical management during that period. The increased variability in time to remission during the pandemic, coupled with the presence of extreme values, suggests greater heterogeneity in clinical evolution during the pandemic period, although the underlying reasons cannot be established from this retrospective study. Similar findings have been reported in other pediatric cohorts studied in relation to the reference period (5, 18, 19). Overall, our findings are consistent with differences in healthcare delivery between the two periods; however, the present study was not designed to establish causal relationships.

A notable finding was the significantly lower frequency of infections associated with disease onset during the pandemic compared with the post-pandemic period. This apparent paradox may be explained by public health measures implemented during the pandemic, including social distancing, mask-wearing, and reduced circulation of non-SARS-CoV-2 respiratory pathogens (20, 21). Notably, no patients had acute SARS-CoV-2 infection at diagnosis, suggesting that the virus did not directly trigger disease onset in our cohort. Instead, the higher frequency of infection-related presentations observed in the post-pandemic period likely reflects renewed exposure to common pathogens following the relaxation of public health restrictions (22). Similar trends have also been reported internationally (23, 24).

Although no statistically significant differences in overall corticosteroid response were observed between the pandemic and post-pandemic cohorts, these comparisons should be interpreted with caution. Corticosteroid resistance is generally established early after treatment initiation, whereas corticosteroid dependence is a time-dependent outcome that requires prolonged follow-up. Because patients diagnosed during the post-pandemic period had substantially shorter follow-up, corticosteroid dependence may have been under-ascertained in this group. Consequently, comparisons of the overall corticosteroid response primarily reflect descriptive observations and should not be interpreted as evidence of true differences in long-term therapeutic evolution. However, the trend toward a lower proportion of corticosteroid-sensitive patients during the pandemic, combined with a lower likelihood of a favorable response, may suggest differences in clinical evolution between the two periods, although the underlying mechanisms cannot be determined from the present data. The correlation between the degree of response to corticosteroids and the time to remission confirms the central role of this parameter as a major prognostic determinant, consistent with the literature (25).

An important contribution of the study is the identification of clinical and biological factors that may be associated with infection-related disease onset and response to treatment. In the exploratory logistic regression analysis, the post-pandemic period, anemia at diagnosis, and weight status were associated with infection-related onset. However, these findings should be interpreted cautiously because of the limited sample size, the relatively small number of events, and the wide confidence interval observed for anemia, indicating uncertainty in the estimated effect size. Therefore, these associations should be considered hypothesis-generating and require confirmation in larger, prospectively validated cohorts. In particular, the observed association between anemia and infection-related onset may reflect a potential role of inflammatory or nutritional status in modulating the immune response (26), although this interpretation remains speculative. Regarding the exploratory analysis of factors associated with corticosteroid response, macroscopic hematuria was the only significant predictor, being associated with an increased risk of unfavorable outcome. This result is consistent with the hypothesis that the presence of hematuria reflects more severe glomerular damage or a different histological substrate than minimally damaged disease – an aspect that is in agreement with the data in the literature regarding the correlation of hematuria - renal injury (27, 28). Low albuminemia also showed a similar trend, suggesting its potential value as a prognostic marker, already validated in extensive reference studies (29).

Analysis of biological parameters did not reveal subtle, statistically significant differences during the pandemic period for most variables. However, significant increases in fibrinogen and complement C4 fraction were noted. These changes suggest a transient activation of the immune response and the coagulation cascade, but do not indicate marked systemic inflammation, given the lack of significant differences in CRP, ESR, or NLR. The increase in fibrinogen, in the absence of significant changes in D-dimers, indicates a procoagulant rather than a consumptive state, which is relevant in the context of the thrombotic risk associated with nephrotic syndrome (30). These observations may reflect indirect effects of systemic stress or behavioral changes during the pandemic. In an exploratory linear regression analysis, CRP emerged as the strongest variable associated with baseline ESR. This finding is consistent with the established biological relationship between ESR and CRP, both being markers of the acute-phase response, and therefore should not be interpreted as evidence of a novel mechanistic association. Rather, the model confirms that ESR variability in this cohort primarily reflects the inflammatory state, while albumin and fibrinogen may provide complementary information. The regression model should nevertheless be interpreted with caution because of the limited sample size (n = 25), which may reduce the stability of coefficient estimates despite satisfactory diagnostic performance. External validation in larger cohorts is warranted. These results are consistent with data from the literature showing that ESR predominantly reflects the effects of acute phase proteins and is closely correlated with CRP, while albumin, as a marker of systemic and nutritional status, has a complementary and indirect role in determining its variation (through its influence on erythrocyte aggregation and plasma protein behavior) (31–33).

Because complement C4 was the only complement component that differed significantly between the pandemic and post-pandemic cohorts, we performed exploratory univariable analyses to evaluate its relationship with selected clinical characteristics. Although numerically higher C4 values were observed among patients with non-corticosteroid-sensitive disease and among those with macroscopic hematuria, none of these comparisons reached statistical significance. Likewise, no significant associations were observed with elevated blood pressure or age at diagnosis. These findings should therefore be considered descriptive and hypothesis-generating, particularly given the limited number of patients with available C4 measurements. Previous studies investigating the relationship between complement activation and corticosteroid response have reported inconsistent results (34–38), and larger prospective studies are required to clarify the potential role of C4 in pediatric idiopathic nephrotic syndrome.

In conclusion, the findings of this study suggest differences in healthcare utilization and clinical evolution between children diagnosed during the pandemic and post-pandemic periods, whereas the biological characteristics of the disease remained largely comparable. The identification of potential clinical and biological factors associated with disease course, such as hematuria, anemia, and weight status, provides preliminary insights into risk stratification. However, these findings should be considered exploratory and require external validation before they can be incorporated into clinical decision-making or predictive models. The interpretation of these data should be done with caution, given several limitations: the relatively small cohort size, the monocentric nature of the study, the retrospective nature of data collection, and the lack of systematic histopathological information - as renal biopsy was performed only in a limited number of clinically selected patients and at variable time points during follow-up, precluding standardized histopathological comparisons. The interpretation of treatment response should also be considered in light of the unequal follow-up duration between the pandemic and post-pandemic cohorts. While corticosteroid resistance is determined relatively early during the disease course, corticosteroid dependence requires prolonged observation and may therefore have been underestimated in patients diagnosed during the later study period. Because of the retrospective design, variable attendance at follow-up visits, and the limited duration of observation for recently diagnosed patients, standardized long-term assessment of treatment evolution was not feasible. Moreover, corticosteroid dependence is not defined by a single uniformly timed event but requires remission, repeated corticosteroid tapering attempts, and subsequent relapses according to accepted definitions. Because these intervals vary according to individual disease course and therapeutic management, standardized time-to-event modelling was not considered methodologically appropriate within the retrospective design of the present study (39–42). Also, socio-economic factors, treatment adherence, or the psychosocial impact of the pandemic were not included, and the microbiological characterization of infections was incomplete. Accordingly, the exploratory regression models presented in this study should be interpreted primarily as hypothesis-generating. Their apparent performance was estimated only in the development dataset and no internal or external validation was performed; therefore, they should not be considered clinically predictive models.

## 5 Conclusions

The present study found similar demographic characteristics and numbers of diagnosed pediatric INS cases between the pandemic and post-pandemic periods. The main differences observed between the two periods included longer hospitalization, a more heterogeneous time to remission, and differences in the frequency of infection-associated onset. Although the median interval between symptom onset and presentation was numerically longer during the pandemic, this difference was not statistically significant. In this cohort, the observed corticosteroid response was associated with subsequent clinical evolution. However, conclusions regarding corticosteroid dependence should be interpreted cautiously because patients diagnosed during the post-pandemic period had substantially shorter follow-up, limiting the assessment of long-term treatment evolution. Differences in fibrinogen and complement C4 levels were observed between the two cohorts, although their clinical significance remains uncertain. Exploratory descriptive analyses showed numerically higher C4 values among patients with non-corticosteroid-sensitive disease and among those with macroscopic hematuria, although these differences did not reach statistical significance. Likewise, no significant association was observed between C4 levels and elevated blood pressure or age at diagnosis. Given the limited number of patients with available C4 measurements, these findings should be considered exploratory and hypothesis-generating. Other clinico-biological factors, such as anemia at diagnosis and weight status, were associated with infection-related onset in the exploratory analysis and may warrant further investigation as candidate markers for future risk stratification. Overall, these findings contribute to a better understanding of the clinical characteristics of pediatric idiopathic nephrotic syndrome diagnosed during and after the pandemic and support further investigation in larger prospective cohorts.

## Data Availability

The original contributions presented in the study are included in the article/**Supplementary Material**. Further inquiries can be directed to the corresponding authors.

## Supplementary material

Supplementary Figure 1 Receiver Operating Characteristic (ROC) Curve for the Binary Logistic Regression Model Predicting Infection-Associated Onset of Nephrotic Syndrome.

Supplementary Figure 2 Assessment of Residual Normality: Q-Q Plot for ESR Linear Regression.

Supplementary Figure 3 Marginal Means Plots of ESR by CRP, NLR, Fibrinogen, and Albumin, with corresponding mean ± SD tables.

Supplementary Figure 4 Receiver Operating Characteristic (ROC) Curve of the logistic regression model for predicting corticosteroid resistance in children with nephrotic syndrome.

Supplementary Figure 5 Adjusted marginal means of time to remission by corticosteroid response, with 95% CI.

Supplementary Table 1 Multiple linear regression results for ESR (N = 25).

Supplementary Table 2 Clinical variables associated with corticosteroid response in pediatric nephrotic syndrome – binomial logistic regression (N = 29).
