# Supplementary material for: Pandemic and post-pandemic shifts in onset and severity of pediatric nephrotic syndrome: a comparative cohort analysis
Source: Front Immunol. 2026 Aug 26;17:1860961. doi: 10.3389/fimmu.2026.1860961 (PMC13553929; doi:10.3389/fimmu.2026.1860961)
Supplement: Supplementary file 1 [file Table1.docx]

**Supplementary Table 1**. Multiple linear regression results for ESR (N = 25)

| **Predictor** | **β (Estimate)** | **Standard Error** | **95% CI** | **t** | **P - value** | **Stand. β** | **VIF** | **ANOVA F (Type III)** | **ANOVA p** |
| --- | --- | --- | --- | --- | --- | --- | --- | --- | --- |
| Intercept | 19.022 | 21.709 | −26.263 – 64.306 | 0.876 | 0.391 | – | – | – | – |
| Fibrinogen (mg/dl) | 0.046 | 0.024 | −0.003 – 0.096 | 1.944 | 0.066 | 0.345 | 1.45 | 3.78 | 0.066 |
| Albumin (g/dl) | 18.364 | 9.566 | −1.589 – 38.316 | 1.920 | 0.069 | 0.396 | 1.96 | 3.69 | 0.069 |
| NLR | −6.397 | 2.102 | −10.781 – −2.013 | −3.043 | 0.006 | −0.579 | 1.67 | 9.26 | 0.006 |
| CRP (mg/L) | 0.535 | 0.173 | 0.174 – 0.895 | 3.096 | 0.006 | 0.522 | 1.31 | 9.58 | 0.006 |
| Model Fit | R: 0.752; R²: 0.565; Adjusted R²: 0.478; RMSE: 15.3  F (df1, df2): 6.50 (4, 20)  p (overall model) 0.002  Cook’s Distance: max = 0.786; mean = 0.0273; median = 0.0132; SD = 0.253  Mahalanobis Distance: max = 5.06; mean = 2.92; median = 3.84; SD = 0.240; n. obs > threshold = 2  Durbin–Watson: 1.92; p = 0.812  Shapiro–Wilk (residuals): 0.926; p = 0.069 | | | | | | | | |

sss
