# Supplementary material for: Pandemic and post-pandemic shifts in onset and severity of pediatric nephrotic syndrome: a comparative cohort analysis
Source: Front Immunol. 2026 Aug 26;17:1860961. doi: 10.3389/fimmu.2026.1860961 (PMC13553929; doi:10.3389/fimmu.2026.1860961)
Supplement: Supplementary file 2 [file Table2.docx]

**Supplementary Table 2.** Clinical variables associated with corticosteroid response in pediatric nephrotic syndrome – binomial logistic regression (N=29)

| Predictor | Codificare | Coeficient (β, logit) | Standard Error | Z | P | Clinical interpretation |
| --- | --- | --- | --- | --- | --- | --- |
| Intercept | – | 4.391 | 4.179 | 1.051 | 0.293 | Log-odds for non-corticosensitivity when all predictors = 0 |
| Gross hematuria | 0 = present, 1 = absent | −4.612 | 2.238 | −2.060 | **0.039** | The presence of hematuria increases the risk of corticosteroid insensitivity |
| Albuminemia at diagnosis (g/dl) | – | −3.302 | 2.338 | −1.412 | 0.158 | Higher albumin levels decrease log-odds of corticosteroid insensitivity |
| Time to remission (days) | – | 0.056 | 0.036 | 1.546 | 0.122 | Longer duration may increase risk, nonsignificant effect |
| Gender | 1 = boy, 2 = girl | 0.467 | 1.231 | 0.379 | 0.705 | Girls have a slightly higher, non-significant risk |
| Correlation of onset with infections | 0 = correlated, 1 = uncorrelated | 0.843 | 1.311 | 0.643 | 0.520 | Correlation with infection at onset does not significantly influence risk |
| Anemia | 0 = present, 1 = absent | −1.408 | 1.568 | −0.899 | 0.369 | Anemia has no significant effect |
| Logic model performance | R² McFadden = 0.415  AIC = 33.1, BIC = 42.7  AUC = 0.887  Specificity = 95.2%, Sensitivity = 75% at cut-off 0.5  Colinearity: VIF < 3 for all predictors  Omnibus χ² (df=6): 15.0, p = 0.020 | | | | | |
